# Nutritional quality and costs of gluten-free products: a case-control study of food products on the Norwegian marked

**DOI:** 10.29219/fnr.v65.6121

**Published:** 2021-03-26

**Authors:** Mari C. W. Myhrstad, Marlene Slydahl, Monica Hellmann, Lisa Garnweidner-Holme, Knut E. A. Lundin, Christine Henriksen, Vibeke H. Telle-Hansen

**Affiliations:** 1 Department of Nursing and Health Promotion, Faculty of Health Sciences, Oslo Metropolitan University, Oslo, Norway; 2 Det Glutenfrie Verksted, Oslo, Norway; 3 K.G. Jebsen Coeliac Disease Research Centre, University of Oslo, Oslo, Norway; 4 Department of Gastroenterology, Oslo University Hospital, Oslo, Norway; 5 Department of Nutrition, Institute of Basic Medical Sciences, University of Oslo, Oslo, Norway

**Keywords:** gluten-free products, gluten-containing products, database, nutritional quality, macronutrients, fiber, price, unhealthy diet

## Abstract

**Background:**

Celiac disease is a chronic autoimmune disease triggered by gluten exposure in genetically predisposed individuals. A life-long intake of a gluten-free (GF) diet is required for its management. Wheat, rye and barley are eliminated in a GF diet and the nutritional adequacy of the diet has been questioned. In Norway, cereals and bread constitute a key role of the diet and are the main source of fiber intake. Gluten restrictions may therefore offer important implications for nutrient adequacy especially linked to fiber intake in people with celiac disease.

**Objective:**

The aim of the study was to investigate the nutritional quality and price of GF products and compare with gluten-containing counterparts available at instead of in the Norwegian market.

**Design:**

The macronutrient content of 423 unique GF products were compared with 337 equivalents with gluten. All products were selected from grocery stores and web-based shops, with the aim of including as many GF products as possible. Listed macronutrients content and price in 11 different food categories were compared to gluten-containing counterparts with Wilcoxon signed rank test.

**Results:**

The GF products contained less protein and fiber, and higher content of saturated fat, carbohydrate and salt compared to the gluten-containing products. The total amount of fat was not different between the groups. A similar pattern was found within several of the food categories. More gluten-containing products met the nutrition claim “high in fiber” (fiber > 6 g/100 g) compared to the GF products. The price of the GF products was higher; ranging from 46%–443% more expensive than the gluten-containing products.

**Conclusion:**

GF products are less nutritious and have a higher price compared to equivalent gluten-containing products. Knowing that an unhealthy diet is the most important risk factor for developing non-communicable diseases, the nutritional quality of a GF diet needs to be addressed and should be improved.

## Popular scientific summary

- The only treatment for celiac disease, is a lifelong gluten-free diet.
- Removing gluten-containing ingredients from the diet may challenge the nutritional quality of the diet.
- The aim was to investigate the nutritional quality and price of gluten-free products at the Norwegian market.
- The gluten-free products are less nutritious and have a higher price compared to equivalent gluten-containing products.
- Following a gluten-free diet should therefore be restricted for those with medical requirements.

Gluten-related disorders including celiac disease (CD), wheat-allergy and non-coeliac gluten sensitivity affects millions of peoples worldwide (1), (2). Celiac disease is a hereditable chronic autoimmune bowel disease and is triggered by gluten exposure and has a worldwide prevalence of around 1% (3), (4). The only treatment for CD and other gluten-related disorders is a lifelong gluten-free (GF) diet (5), (6). Gluten is a group of proteins termed prolamins and glutelins found in cereal grains such as wheat, rye and barley (7). Gluten is therefore mostly found in bread, cereals and pasta (8). Gluten proteins have unique viscoelastic and adhesive properties giving the puffy and chewy texture in dough. Furthermore, these properties combined with its relative low cost are reasons why gluten is used as a stabilizing agent by the food industry in a variety of products ranging from seasoning, marinades and sauces to ice cream (9).

During the last decades, the popularity of GF products has increased in the general population (10). This is probably due to a public perception that a GF diet is health beneficial, and GF products are today widely used also by people without a gluten-related disorder diagnosis (8). Even so, there is little evidence supporting this belief and the nutritional adequacy of a GF diet has been questioned (11). GF products are normally made with starches and or refined flours characterized by a low content of fiber (8), (12). Furthermore, studies indicate that GF products usually have a higher carbohydrate and fat content than their gluten containing counterparts and the GF products is particularly high in saturated fatty acids (13), (14). Indeed, a GF diet has been characterized by an altered intake of macronutrient and micronutrient and a lower intake of dietary fiber compared to a regular diet containing gluten (8), (12), (15), (16). The nutritional quality of GF food in Norway is currently not known.

High intake of dietary fiber provides health benefits and reduces the risk of developing non-communicable diseases such as diabetes type 2, colon cancer and cardiovascular disease (17), (18). In Norway, gluten-containing cereals and grains have an important role in the diet and represent the main source of dietary fiber (19), (20). The Norwegian health authorities recommend a daily intake of 70–90 g of whole grain or whole meal flour, also for people with CD. However, this is especially challenging in a GF diet were gluten-containing cereals and grains high in fiber are removed. Furthermore, higher costs of GF products are familiar challenges for persons with CD. Data indicates that most GF products are several times more expensive than gluten-containing food (21). Since a GF diet is medicine for people with CD, several countries offer a basic allowance to compensate for the expenses. Whether the financial support is adequate requires updated information on price of GF products compared to gluten-containing equivalents. Furthermore, since price is a driving force of our food choices (22), (23) it is important to get more knowledge about the cost of a GF diet.

The aim of the study was therefore to investigate the nutritional quality of GF products and compare them with gluten-containing counterparts available in the Norwegian market. In addition, the study aims to investigate whether GF products are more expensive than similar gluten-containing products.

## Materials and methods

### Grocery stores and product selection

A database containing information of the nutrient content and price of GF and gluten-containing products from the Norwegian grocery stores were developed during autumn 2019. Three web-based (Kolonial.no, Meny.no, and Allergikost.no), and five grocery stores in the area of Oslo and Viken (Meny Bryn, Meny Sandvika, Jacobs Utvalgte Majorstuen, Coop Obs Haugenstua, Rema 1000 kanalveien) were visited during September to October 2019. The stores were chosen based on the assortment of GF products available as we aimed to cover as many GF products in the Norwegian market as possible. A supplementary recording was performed after new products were launched for retail sale, post autumn 2019.

Information on nutrient content including energy, total fat, saturated fat, protein, carbohydrate, sugar, fiber and salt in addition to price were recorded for all the GF and gluten-containing products. If available, the content of mono- and polyunsaturated fat were also recorded. In the grocery stores a photograph of the nutrition facts panel and the package front were taken and the price noted.

Naturally GF foods such as rice, meat, fruits and vegetables were not included in the study. Items were considered GF if the product packaging or description included a cross grain mark, or some other GF declaration and the products were categorized as free from gluten by the grocery store. To ensure that all products from the grocery stores were included, the shelves were assessed systematically from top to bottom.

For every GF product, a comparable gluten-containing product whose description most closely matched the type of food and intended use was chosen. The gluten-containing products met the dietary guidelines or had the ‘Keyhole’ symbol, if possible. The keyhole symbol is a voluntary Nordic label for food; products with the Keyhole contain more dietary fiber, less saturated fat, less salt and/or less sugar compared to other foods of the same type (24). Only gluten-containing bread with the Norwegian ‘bread scale’ was chosen, preferably the ones with 4/4 or 3/4 squares on the scale. The ‘bread scale’ is a labeling method and indicates the percentage of wholegrain in the bread. A bread scale with 3/4 or 4/4 squares contains 51–75.9% and 76–100% wholegrain or whole-meal flour, respectively. When several gluten-containing products were available, one not sampled earlier were chosen to increase the number of gluten-containing products followed by the least expensive gluten-containing alternative. In cases of only one available product, the gluten-containing product was matched with several different GF products. The method used was inspired from previous studies with a similar aim (13), (14).

The GF products were categorized into 11 different groups (Table 1), based on intended use and regular food-categories from web-based grocery stores. A brief description of products included in the categories is shown in Table 1. The final database consisted of 423 unique GF products and 337 unique gluten-containing products. In some cases, the same gluten-containing product had to match more than one GF product, as it was the only similar product available.

**Table 1:** Description of GF products included in the 11 food categories

Food category	Description of included products
Bread	All sorts of bread, ciabatta, baguettes, paninis, rolls and sandwich bread
Cripsbread	All sorts of crispbread
Cereals	All oat products, muesli, granola and different types of breakfast flakes.
Flour-mix	Mixes of flours without added seasonings and sugar
Baking mix	Flour products with additives like sugar, yeast and different seasonings, including mixes for waffles, buns, cakes, cookies and similar products
Flour	Flours available on the Norwegian market, including Buckwheat, millet, quinoa, chickpea, rice, maize, soy, Locust bean gum,
Pasta	All sorts of pasta, spaghetti, macaroni, fusilli, pasta screws, penne, lasagna plates and tagliatelle
Dinner products	Products that is used for dinner, such as soups, dinner kit, sauces and marinades (except pizza and pasta)
Pizza	Frozen pizza and pizza crusts
Cakes	Sweet cakes
Snacks	Cookies, biscuits, a broad spectrum of energy bars, crackers and chocolate

GF, Gluten-free.

### Data analysis

Non-duplicate GF products were treated as ‘cases’, while gluten-containing counterparts were treated as ‘controls’. All data are reported in median nutrient content per 100 g of GF versus gluten-containing products, and compared using Wilcoxon rank sum test due to small sample-size. All analyses were done in SPSS Statistics (version 26.0.). The level of significance was set at *P* < 0.05 for all analyses.

## Results

The nutritional content of all the unique GF products (*n* = 423) were compared to similar gluten-containing products (*n* = 337) in paired analyses, and is presented in Table 2. The amount of saturated fat (*P* < 0.01), carbohydrate (*P* < 0.01) and salt (*P* < 001) was higher and the amount of fiber (*P* < 0.01) and protein (*P* < 0.01) lower in GF products compared to gluten-containing products. There were no significant differences in the amount of sugar and total fat between the two groups. A similar pattern was observed in several of the different food categories, as presented below.

**Table 2:** Energy and nutrient content in GF and comparable gluten-containing products

Nutrients g/100 g	GF median (25th–75th)	G median (25th–75th)	*P*
	*n* = 423	*n* = 337	
Calories (kcal)	359 (276–415)	350 (270–390)	***<0.01***
Total fat	5.5 (2.0–15.0)	5.6 (2.3–12.0)	0.279
Saturated fat	1.0 (0.5–3.8)	0.9 (0.4–2.8)	***<0.01***
Carbohydrate	61.0 (44.0–74.0)	57.7 (42.2–65.0)	***<0.01***
Protein	5.8 (3.5–8.5)	9.5 (7.3–12.0)	***<0.01***
Fiber	4.4 (2.2–7.5)	6.0 (3.0–8.9)	***<0.01***
Sugar	3.5 (1.1–14.8)	3.3 (2.0–11.0)	0.717
Salt	0.8 (0.3–1.2)	0.7 (0.1–1.0)	***<0.01***

GF, Gluten-free; G, Gluten-containing.

Data is given as median (25th–75th). *P*-values indicate differences between GF and comparable G products calculated with Wilcoxon signed rank test.

The level of significance was set at *P* < 0.05 and are indicated in bold italic.

### Bread, crisp bread and cereals

The nutritional content of GF bread (*n* = 66), crisp bread (*n* = 25) and cereals (*n* = 62) were compared to similar gluten-containing products (Table 3). Higher amounts of total fat (59%, *P* < 0.01), saturated fat (80%, *P* < 0.01), carbohydrate (6%, *P* = 0.03) and salt (11%, *P* < 0.01) and less protein (53%, *P* < 0.01) were found in GF bread compared to gluten-containing bread. GF crisp bread contained more carbohydrate (26%, *P* < 0.01) compared to gluten-containing counterparts, and less protein (56%, *P* < 0.01) and fiber (49%, *P* < 0.01), respectively. Furthermore, GF cereals contained more carbohydrates (6%, *P* = 0.03), sugar (3%, *P* < 0.01) and salt (56%, *P* = 0.05) than comparable gluten-containing products. In addition, less fiber (32%, *P* < 0.01) was noted in the GF compared to the gluten-containing cereals.

**Table 3:** Energy and nutrient content of GF and comparable gluten-containing bread, cereals and crisp bread products

Nutrients g/100 g	Bread			Cereals			Crisp bread		
	GF (*n* = 66) median (25th–75th)	G (*n* = 57) median (25th–75th)	*P*	GF (*n* = 62) median (25th–75th)	G (*n* = 45) median (25th–75th)	*P*	GF (*n* = 25) median (25th–75th)	G (*n* = 23) median (25th–75th)	*P*
Calories (kcal)	267 (241–294)	251 (240–261)	***0.01***	382 (363–420)	370 (363–392)	0.28	366 (359–427)	360 (340–449)	0.35
Total fat	5.9 (3.7–8.7)	3.7 (2.4–6.0)	***<0.01***	6.6 (2.0–15)	6.7 (3.5–7.8)	0.21	4.5 (3.1–16.7)	5.4 (2.5–21.6)	0.41
Saturated fat	0.9 (0.6–1.1)	0.5 (0.4–0.8)	***<0.01***	1.1 (0.5–3.7)	1.3 (0.8–2.0)	0.14	1.2 (0.6–2.8)	0.8 (0.5–2.8)	0.06
Carbohydrate	44.0 (37.9–50.5)	41.7 (38.0–43.9)	***0.03***	65.5 (56.6–80.0)	62.0 (58.0–69.0)	***0.03***	72.0 (50.8–74.1)	57.3 (39.7–61.4)	***0.01***
Protein	4.1 (2.4–6.1)	8.8 (8.4–11.0)	***<0.01***	9.1 (6.7–12.0)	10.9 (9.0–12.0)	0.12	5.3 (4.9–9.4)	12.0 (10.2–17.1)	***<0.01***
Fiber	6.3 (4.7–8.1)	6.0 (5.5–6.8)	0.71	6.7 (3.7–9.0)	9.8 (8.0–12.0)	***<0.01***	8.0 (7.4–10.6)	15.7 (12.7–20.0)	***<0.01***
Sugar	2.7 (1.0–3.9)	2.2 (1.7–3.1)	0.52	9.0 (5.6–21.5)	8.8 (4.4–12.0)	***<0.01***	2.8 (1.6–3.8)	2.0 (1.5–2.3)	0.08
Salt	1.0 (0.9–1.3)	0.9 (0.7–1.0)	***<0.01***	0.5 (0.2–0.8)	0.32 (0.2–0.7)	***0.05***	1.3 (1.1–1.5)	1.1 (1.0–1.3)	0.09

GF, Gluten-free; G, Gluten-containing.

Data is given as median (25th–75th). *P*-values indicate differences between GF and comparable gluten-containing products calculated with Wilcoxon signed rank test.

### Flour-mix, baking-mix and flour

The nutritional differences were less pronounced across the flour-mix, baking-mix and flour products (Table 4). The flour-mix category consisted of 12 mixes of clean flours without added seasonings and sugar. No nutritional differences in the content of fat, carbohydrate, fiber, sugar or salt were found except for a lower protein level (71%, *P* < 0.01) in the GF compared to gluten-containing flour-mix. Twenty-five GF baking mix products were included in the current study. The carbohydrate content was higher (35%, *P* < 0.01), whereas the total fat and protein content was 74% (*P* < 0.01) and 38% (*P* < 0.01) lower in the GF compared to similar gluten-containing baking-mix, respectively. We were able to identify 16 GF flours at the Norwegian market. When comparing to similar gluten-containing clean flours, no nutritional differences related to macronutrients and fiber were evident except for a higher level of salt in GF products.

**Table 4:** Energy and nutrient content of GF flour-mix, baking-mix and flour products and comparable gluten-containing

Nutrients g/100 g	Flour-mix			Baking-mix			Flour		
	GF (*n* = 12) median (25th–75th)	G (*n* = 6) median (25th–75th)	*P*	GF (*n* = 25) median (25th–75th)	G (*n* = 14) median (25th–75th)	*P*	GF (*n* = 16) median (25th–75th)	G (*n* = 16) median (25th–75th)	*P*
Calories (kcal)	340 (295–340)	338 (329–377)	0.24	351 (345–374)	338 (311–355)	***0.01***	353 (331–366)	329 (324–340)	0.22
Total fat	1.1 (0.5–2.3)	2.2 (1.6–6.5)	0.07	1.9 (0.9–8.3)	7.3 (1.6–14.3)	***<0.01***	3.0 (1.6–9.0)	2.5 (1.9–4.1)	0.20
Saturated fat	0.3 (0.2–0.5)	0.4 (0.3–1.1)	0.10	0.6 (0.4–2.0)	1.2 (0.4–1.9)	0.78	0.6 (0.3–1.0)	0.3 (0.3–0.9)	0.31
Carbohydrate	74.5 (51.1–82.3)	63.1 (62.5–67.9)	0.53	72.8 (58.5–80.0)	54 (43.1–67.9)	***<0.01***	64.7 (10.9–70.7)	58.9 (54.1–66.5)	0.96
Protein	3.2 (1.3–5.9)	11.2 (11.2–11.4)	***<0.01***	4.0 (2.6–7.0)	6.5 (5.2–11.2)	***<0.01***	11.2 (8.1–22.0)	11.3 (11.1–13.1)	0.57
Fiber	6.7 (3.3–8.8)	7.4 (3.6–10.8)	0.39	2.1 (1.2–4.0)	3.6 (0.8–3.6)	0.81	6.9 (3.8–15.0)	10.7 (4.4–12.5)	0.86
Sugar	1.9 (0.5–3.6)	2 (1.1–2.7)	0.88	8.0 (1.9–34.6)	6.7 (2.7–21.2)	0.42	0.8 (0–1.8)	2.2 (1.4–2.7)	0.28
Salt	0 (0–0.6)	0 (0–0)	0.07	0.6 (0.3–1.0)	0.5 (0–0.9)	0.52	0 (0–0.03)	0 (0–0)	***0.04***

GF, Gluten-free; G, Gluten-containing.

Data is given as median (25th–75th). *P*-values indicate differences between GF and comparable gluten-containing products calculated with Wilcoxon signed rank test.

### Pasta, dinner products and pizza

The category of pasta included 45 GF products (Table 5). GF pasta contained more carbohydrate (17%, *P* < 0.01) and less total fat (10%, *P* < 0.01), protein (46%, *P* < 0.01), fiber (66%, *P* < 0.01) and sugar (78%, *P* < 0.01) when compared to similar gluten-containing products (Table 5). In the category dinner products, 56 products were included (Table 5). In this category more salt (25%, *P* < 0.01) and less total fat (21%, *P* < 0.01) and protein (53%, *P* < 0.01) were noted compared to gluten-containing counterparts. No nutritional differences except for the lower protein content (46%, *P* < 0.01) in 11 GF pizza compared to similar gluten-containing counterparts were found (Table 5).

**Table 5:** Energy and nutrient content of GF pasta, pizza and dinner products and comparable gluten-containing products

Nutrients g/100 g	Pasta			Dinner products			Pizza		
	GF (*n* = 45) median (25th–75th)	G (*n* = 31) median (25th–75th)	*P*	GF (*n* = 56) median (25th–75th)	G (*n* = 48) median (25th–75th)	*P*	GF (*n* = 11) median (25th–75th)	G (*n* = 11) median (25th–75th)	*P*
Calories (kcal)	354 (350–360)	350 (347–350)	***<0.01***	206 (86–288)	201 (103–286)	***0.05***	242 (201–274)	266 (220–280)	0.114
Total fat	1.8 (1.1–2.2)	2.0 (2.0–2.5)	***<0.01***	3.7 (0.7–5.9)	4.7 (1.5–7.3)	***<0.01***	5.9 (2.9–8.1)	4.9 (2.8–8.4)	0.859
Saturated fat	0.5 (0.2–1.0)	0.5 (0.4–0.5)	0.23	0.7 (0.1–1.4)	0.8 (0.2–1.5)	0.06	1.0 (0.5–4.2)	0.9 (0.4–2.8)	0.859
Carbohydrate	76.0 (72.6–78.2)	65 (64–67)	***<0.01***	33.4 (7.8–49.9)	31.9 (11.6–45.2)	0.218	48.2 (25.0–56.0)	43.0 (25.0–48.0)	0.139
Protein	7 (6.4–8.5)	13.0 (12.0–14.0)	***<0.01***	3.9 (1.6–8.9)	8.3 (3.9–10.0)	***<0.01***	4.6 (3.5–7.5)	8.5 (8.1–11.0)	***<0.01***
Fiber	2.4 (1.6–3.0)	7.0 (6.8–8.2)	***<0.01***	1.7 (0.3–5.3)	3.7 (0.7–6.8)	0.499	3.3 (2.1–3.8)	2.0 (0–2.2)	0.068
Sugar	0.7 (0.4–1.0)	3.2 (3.0–3.5)	***<0.01***	2.2 (1.0–4.3)	2.5 (1.5–3.8)	0.792	2.2 (1.4–3.5)	2.5 (1.5–3.3)	0.929
Salt	0.01 (0–0.1)	0 (0–0.01)	***0.03***	1.0 (0.8–1.3)	0.8 (0.7–1.0)	***<0.01***	1.1 (0.8–1.5)	1.0 (0.9–1.5)	0.656

GF, Gluten-free; G, Gluten-containing.

Data is given as median (25th–75th). *P*-values indicate differences between GF and comparable G products calculated with Wilcoxon signed rank test.

### Cakes and snacks

No nutritional differences related to fat, protein, carbohydrate, fiber, sugar or salt content were found when 14 sweet GF cakes were compared to similar gluten-containing cakes (Table 6). Eighty-eight unique GF snack products were identified in the Norwegian market and included in the analyses (Table 6). Altogether the GF products contained more saturated fat (66%, *P* = 0.014), carbohydrates (4%, *P* < 0.01) and fiber (3%, *P* < 0.01) than similar gluten-containing products, respectively. Thirty-two percent (*P* < 0.01) less protein was noted in GF compared to gluten-containing snack products.

**Table 6:** Energy and nutrient content of GF cakes and snacks and comparable gluten-containing products

Nutrients g/100 g	Cakes			Snacks		
	GF (*n* = 14) median (25th–75th)	G (*n* = 13) median (25th–75th)	*P*	GF (*n* = 88) median (25th–75th)	G (*n* = 76) median (25th–75th)	*P*
Calories (kcal)	419 (318–443)	387 (350–446)	0.367	462 (422–501)	470 (405–500)	0.095
Total fat	19 (9.0–24.2)	17.9 (12.1–23.5)	0.937	21.0 (13.8–25.0)	19 (11.5–25.0)	0.126
Saturated fat	5.5 (2.8–10.3)	5.9 (3.0–8.3)	0.388	9.8 (3.7–13.0)	5.9 (2.6–12.0)	***0.014***
Carbohydrate	51.0 (47.8–61.2)	54.7 (45.9–56.0)	1.00	65.3 (59.5–72.0)	62.5 (56.2–67.8)	***<0.01***
Protein	4.4 (3.6–5.1)	5.3 (5.0–5.8)	0.062	4.9 (3.1–7.7)	7.2 (5.8–9.4)	***<0.01***
Fiber	2.7 (0.7–4.2)	2.7 (2.5–10.5)	1.00	3.4 (2.3–5.2)	3.3 (2.3–5.8)	***<0.01***
Sugar	29.0 (15.7–41.8)	34.7 (23.1–38.0)	0.784	26.7 (4.9–32.7)	22.6 (7.0–36.1)	0.151
Salt	0.4 (0.3–0.9)	0.6 (0.3–0.8)	0.906	0.7 (0.4–1.3)	0.7 (0.4–1.3)	0.288

GF, Gluten-free; G, Gluten-containing.

Data is given as median (25th–75th). *P*-values indicate differences between GF and comparable C products calculated with Wilcoxon signed rank test.

### Price difference between GF and gluten-containing products

Price per kilo of all the unique GF products were compared to the gluten-containing counterparts and the results for all products and within each food category are presented in Table 7. Altogether, GF products were 113% (*P* < 0.01) more expensive than comparable gluten-containing products. The GF products were significantly higher priced compared to gluten-containing products in all categories except for the cakes, ranging from 46 to 443% more expensive. The highest differences in price were identified in the flour-mix and flour categories, where GF products cost 421 and 443% more than similar gluten-containing products, respectively.

**Table 7:** Price per kilo of GF and comparable gluten-containing products

Price per kg	GF (*n* = 423) median (25th–75th)	G (*n* = 337) median (25th–75th)	*P*	% Difference*
All products	170 (111–232)	80 (45–151)	*<0.01*	113
Bread	133 (104–189)	47 (30–65)	***<0.01***	183
Cereals	136 (111–179)	80 (55–129)	***<0.01***	70
Crispbread	207 (186–241)	142 (68–177)	***<0.01***	46
Flour-mix	73 (59–95)	14 (10–33)	***<0.01***	421
Baking-mix	98 (80–113)	44 (10–86)	***<0.01***	123
flour	125 (77–154)	23 (15–42)	***<0.01***	443
Pasta	106 (79–178)	49 (42–62)	***<0.01***	116
Dinner products	209 (161–299)	109 (68–238)	***<0.01***	92
Pizza	155 (115–173)	100 (54–115)	***<0.01***	55
Cakes	251 (174–323)	125 (104–216)	0.116	101
Snacks	293 (213–406)	200 (132–236)	***<0.01***	47

GF, Gluten-free; G, Gluten-containing.

Data is given in Norwegian kroner (NOK) with median value per kg product and 25th and 75th. 1 NOK = 0095 EURO (nov-2020).

* % increase in price from gluten-containing products.

### High fiber content in GF products compared to gluten-containing products

Finally, we investigated the percentage of GF products that met the nutrition claim related to high fiber content given by the European Food Safety Authority (EFSA) (www.EFSA.europa.eu) and compared them to the gluten-containing products. According to EFSA, food products high in fiber may only be claimed if the product contains at least 6 g of fiber/100 g. Altogether, the fiber content was specified for 353 GF and 360 gluten-containing products, respectively. Across all GF products 38.2% were classified as high in fiber whereas 50.6% of the gluten-containing products met the same criteria for a nutritional claim (Table 8). The percentage of GF products with a high fiber content were thereafter calculated within selected food categories (bread, crisp bread, cereals, flour-mix, flour and pasta) known to contribute to the dietary fiber intake. The results show that the percentage of products that met the nutrition claim ‘high in fiber’ was higher for the gluten-containing products compared to the GF products in all the selected categories (Table 8).

**Table 8:** Percentage of the GF and gluten-containing products that classify as high in dietary fiber and meet the Nutrition Claim given by the European Food Safety Authority (www.EFSA.europa.eu) across all categories and within the selected category

Food category	GF products high in fiber (fiber > 6 g/100 g)	G products high in fiber (fiber > 6 g/100 g)
All categories*	38.2%	50.6%
Bread	54.5%	55.4%
Cereals	65.6%	80%
Crips bread	88%	100%
Pasta	12.2%	67.9%
Flour mixes	58.3%	66.7%
Flour	53.8%	75%

GF, Gluten-free; G, Gluten-containing.

* Results for all products within the all the categories (11) are shown. The fiber content was specified for 353 GF and 360 gluten-containing products, respectively.

## Discussion

The current study clearly shows that GF products compared to equivalent gluten-containing products contain less protein and fiber, and more carbohydrate, saturated fat and salt. Furthermore, GF compared to gluten-containing products are more expensive. To our knowledge, this is the first study comparing GF products at the Norwegian market with gluten-containing counterparts.

The results are in line with similar studies performed in other countries and may explain the altered intake of nutrients and especially lower intake of fiber in people eating a GF diet compared to a regular diet (8), (13), (14), (16), (25), (26).

In the present study, the fiber content was significantly lower across all the GF products and in the categories of cereals, crisp bread and pasta compared to similar gluten-containing products. Similar results were found in a study from Canada where GF staple food contained less fiber compared to gluten-containing staple food (13). In a study conducted in the United Kingdom, the GF products were also more likely to be lower in fiber than regular foods and especially pasta products (14). Of interest is our finding that the fiber content did not differ between GF and gluten-containing bread and the percentage of bread that were classified as high in fiber (>6 g/100 g) was similar in bread with and without gluten. This is in accordance with findings from other countries were no differences in fiber content existed between GF and gluten-containing bread (14), (27). Even so, across all food categories and for cereals, crisp bread, pasta, flour-mix and flour a higher percentage of the gluten-containing products were classified as high in fiber (>6 g/100 g) as compared to the GF products. As the dietary fiber intake in the western world is below the dietary recommendations (28), this may be a cause for concern regarding GF diet and adequate fiber intake.

In addition to the lower fiber content, lower protein content was also observed across the GF compared to similar gluten-containing products and was evident in most of the categories, except for cereals, flour and cakes. This has also been observed in several of the studies discussed above (14), (25), (26), (29). In line with our results, Jamieson et al. found that the protein content was lower in all categories except for flour products (13). The lower protein content in the GF products may indicate that the gluten protein impacts the overall protein content in gluten-containing food. The key contributors to dietary protein intake (meat, fish, eggs and dairy products) is naturally free of gluten and the lower protein content in the GF products may therefore not offer a concern regarding adequate protein intake. However, those who are restricted to a GF diet and only consume protein from vegan sources may potentially be at risk of an inadequate protein intake.

In the present study, the content of saturated fat was significantly higher across all GF products compared to the gluten-containing products. Analyzing the categories separately, bread and snacks were the only categories with significantly higher amounts of saturated fat compared to the gluten-containing counterparts. This has also been observed in other studies using information from both food labels and from chemical analysis of GF food (14), (26), (30). This may be caused by differences in the fat content of the GF ingredients used, or that fat and especially saturated fat is added to improve food texture (31). The intake of saturated fatty acids is recommended to be less than 10% of total energy intake (32). Moreover, the exchange of saturated fat in the diet with mono- and polyunsaturated fat is preferable as unsaturated fat is associated with reduced risk of cardiovascular disease (33). Celiac disease has been associated with a modest increased risk of cardiovascular disease (34) and it is therefore not desirable that GF products provide more saturated fat than comparable gluten-containing products.

Overall GF products contained more salt compared to gluten containing benchmark products, especially in bread, cereals, flours and dinner products. This has also been observed in GF products in other countries (13), (14), (26), (30). There is a strong association between salt intake and increased risk of high blood pressure (35) and high intake of sodium is one of the leading dietary risk factors for deaths worldwide (36). The World Health Organization therefore recommends that the intake of salt should be limited to maximum 5 g/day (37). In Norway the consumption of salt is above the recommendation (19), and a private-public partnership has been established, with the goal to reduce the salt consumption with 30% by 2025. The partnership has defined goals on the amount of salt in given food categories (38). Interestingly, GF products are not included in these goals and GF bread and crisp bread are higher in salt than recommended for gluten-containing products. Taken together, people following a GF diet are in danger of consuming more salt than recommended and more salt than the general population. In the present study, the price of GF products compared to gluten-containing products was 113% higher across all products and in all the food categories. The largest difference in price was observed for flour-mixes and flours. Similar results have been reported in other countries (21), (25), (39). A study of the economic burden of a GF diet in the United States of America found that GF products were more expensive (overall 183%) and that GF products from mass-market producers were 139% more expensive than wheat based versions of the same products (21). This is probably due to higher cost of ingredients and a smaller sales market for the GF products compared to the gluten-containing food products. In Norway, the government recently reduced the financial support to persons with CD by more than 50% (40). Our data indicate indeed that the economic burden of following a GF diet is higher compared to a regular diet. Given that GF food is medicine for persons with gluten-related disorders a reduction in the financial support by the government is unfortunate, and the consequences related to health needs to be addressed.

Interestingly, data from Canada indicates that 1/3 of the population sought for GF products. Of these, only 7% had medical reasons for consuming GF products and the remaining either perceived GF food to be a healthier option, or had a family member requiring a GF diet (41). Our data and similar studies from other countries do not support the notion of GF food as a healthier option. Knowing that an unhealthy diet is the most important risk factor for developing non-communicable diseases, the food industry has a responsibility to improve their products together with national policy makers and health authorities. The present study clearly shows that there are variations in the products regarding the nutritional content. A GF diet within the recommendations may therefore require nutritional skills that are not necessarily present among the average consumers. Taken together, a GF diet should be restricted only to those with medical requirements, in close follow-up by clinical nutritionists or dieticians.

In the current study we aimed to include most of the GF products in the Norwegian market. As only GF products available at the time of the collection were included, some seasonal products or products out-of-stock may have been missed. The data collection was however, preformed over several weeks in an attempt to make a complete database of GF products available during the collection period. For every GF product a comparable gluten-containing product, of which the usage and type of food that most closely matched the GF product, was chosen as a control. If possible, the gluten-containing control product met the dietary guidelines or had the ‘Keyhole’ symbol. In some cases, the gluten-containing product had to be matched with more than one GF product, since it was the only one available that met the recommendations. This selection strategy was chosen as the intention was to examine how the nutritional content in GF products diverged from healthy gluten-containing counterparts in addition to comparing data with similar studies in other countries. Another selection strategy regarding gluten-containing control products would most certainly have affected the results. Furthermore, the choice of categories was based on intended use and regular food-categories from the web-based grocery stores. The number of products in each category varies from 11 to 88 products. In categories with fewer products, the estimates may be less reliable than in the categories with more products. Some of the categories such as snacks contain a large variety of products and hence also the nutritional content within the category will vary. Nevertheless, the findings provide a broad insight into the Norwegian GF landscape. The nutritional content of the foods reported would be consistent across Norway due to federal regulations for nutrition packaging and fortification. In addition, many GF products are imported from other countries and the results could therefore, to some extent be generalized to other countries.

In conclusion, GF products are less nutritious and have a higher price compared to gluten-containing products. Following a GF diet should therefore be restricted for those with medical requirements. The consequences of following a GF diet over time needs to be addressed and the food industry together with national policy makers has a responsibility to improve their products.

## Conflict of interest and funding

The study was performed in collaboration with the food industry (Det Glutenfrie Verksted) represented by Monica Hellmann. Mocica Hellmann was not involved in the collection or analysis of the data. Mari C. W. Myhrstad are involved in projects with research grants from industrial partners and has received research fund from Mills AS, none of which are related to the content of this manuscript. Vibeke H. Telle-Hansen is involved in projects with several industrial partners and has received a research fund from Mills AS, all of which is unrelated to the content of this manuscript. The authors have not received any funding or benefits from industry or elsewhere to conduct this study and declare no conflict of interest.
